# Assistive Relaxation Therapy for Older Adults With Insomnia and Mild Cognitive Impairment: A Pilot Study

**DOI:** 10.1093/geroni/igaa057.1519

**Published:** 2020-12-16

**Authors:** Miranda McPhillips, Junxin Li, E John Ward III, Nalaka Gooneratne

**Affiliations:** 1 University of Pennsylvania, Philadelphia, Pennsylvania, United States; 2 Johns Hopkins School of Nursing, Baltimore, Maryland, United States; 3 University of Pennsylvania, School of Medicine, Philadelphia, United States

## Abstract

Insomnia symptoms are prevalent in older adults with mild cognitive impairment (MCI) and can pose treatment challenges. Our objective was to test the preliminary efficacy of tablet-based assistive relaxation therapy (ART) to improve insomnia symptoms in community-dwelling older adults with MCI. ART involves breath-based relaxation techniques coupled with a physical anchoring task to redirect thoughts and disengage from pre-sleep anxiety-provoking cognitions. Using a pilot randomized controlled non-crossover design, 20 participants recruited from one urban adult day center were allocated in a 1:1 ratio to intervention or education only control group for a treatment period of two weeks. Our final sample (n=20) was balanced on all demographic and clinical variables and consisted of Black (100%), female (75%), older adults (mean age 68.85 ± 7.29) with mean Montreal Cognitive Assessment scores of 21.2 ± 2.48. All participants at baseline had insomnia symptoms (mean Insomnia Severity Index (ISI) score 15.8 ± 3.78) and poor sleep quality (mean Pittsburgh Sleep Quality Index (PSQI) score 12.95 ± 0.70); half had daytime sleepiness (Epworth Sleepiness Scale (ESS) score 10.15 ± 1.07). Compared to baseline, participants improved on ISI (9.83 ± 1.32; p=0.0002), PSQI (9.11 ± 1.02; p=0.0016) and ESS (8.17 ± 0.86; p=0.08). The intervention group had statistically significant mean change scores on ISI compared to the control (-7.5 ± 1.37 vs. -3.88 ± 1.48; p=.0461). There were no statistically significant between group differences on PSQI or ESS. Our preliminary results suggest ART therapy is an effective treatment for insomnia symptoms in older adults with MCI.

